# Effects of B Vitamins on Homocysteine Lowering and Thrombotic Risk Reduction—A Review of Randomized Controlled Trials Published Since January 1996

**DOI:** 10.3390/nu17071122

**Published:** 2025-03-24

**Authors:** Mengyan Li, Ruodi Ren, Kunkun Wang, Shan Wang, Allison Chow, Andrew K. Yang, Yun Lu, Christopher Leo

**Affiliations:** 1 Brigham and Women’s Hospital, Boston, MA 02115, USA; meli@bwh.harvard.edu; 2 College of Pharmacy, University of Minnesota, Minneapolis, MN 55415, USA; renruodicc@gmail.com; 3 Fairbanks Memorial Hospital, 340 Cowles Street, Fairbanks, AK 99701, USA; vector0318@gmail.com; 4 NYU Langone Hospital–Long Island, Mineola, NY 11501, USA; shan.wang@nyulangone.org; 5 College of Arts and Science, New York University, New York, NY 10012, USA; ayc8022@nyu.edu; 6 Dartmouth College, Hanover, NH 03755, USA; andrew.k.yang.21@dartmouth.edu; 7 Department of Pharmacy, Hennepin Healthcare System, Minneapolis, MN 55415, USA; 8 Duke Raleigh Hospital, a Campus of Duke University Hospital, School of Medicine, Duke University, Durham, NC 27708, USA

**Keywords:** vitamin B, homocysteine, thromboembolic risk, thrombosis, randomized controlled trials, pyridoxine, folic acid, folate, cobalamin

## Abstract

Homocysteine is an amino acid derived from methionine which is metabolized via vitamin B_6_ (pyridoxine)- and vitamin B_12_ (cobalamin)-dependent pathways. Supplementation of B vitamins has been shown to effectively reduce plasma homocysteine levels. Previous research has also demonstrated an association between lower plasma homocysteine levels and decreased risk of myocardial infarction, stroke, and venous thromboembolism. However, whether supplementation of B vitamins is associated with risk reduction in thromboembolic events and confers clinical benefits remains inconclusive. This review examines clinical trials published over the past 29 years to assess the effects of B vitamin supplementation on thrombotic risk reduction and homocysteine metabolism. The findings from these studies are inconsistent, and the impact of B vitamins on thrombosis prevention remains uncertain. Given the conflicting evidence, further clinical and translational research is necessary to clarify the role of B vitamin supplementation in thrombosis risk reduction.

## 1. Introduction

**Figure 1:**
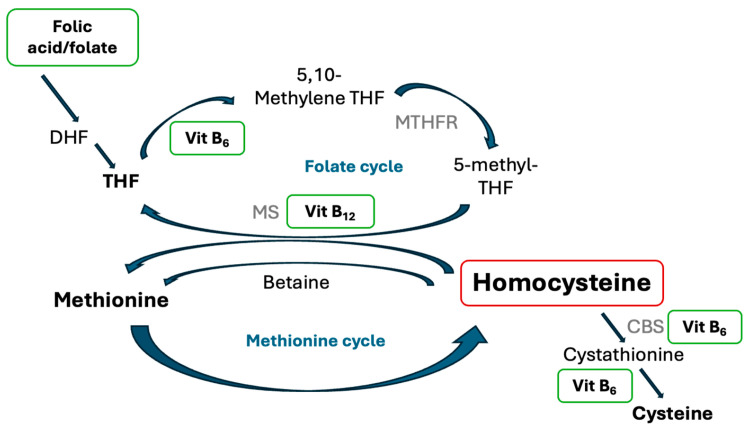
Overview of homocysteine metabolism and the role of folic acid, vitamin B_12_, and vitamin B_6_. Homocysteine can be metabolized through the following three ways: homocysteine can be remethylated to methionine by methionine synthase (MS) with vitamin B_12_ as the cofactor; homocysteine can be converted to cysteine via vitamin B_6_ and cystathionine β-synthase (CBS), a vitamin B_6_-dependent enzyme; or homocysteine can be remethylated to methionine by betaine, a choline derivative. The process of homocysteine remethylation to methionine requires a methyl group derived from 5-methyl-tetrahydrofolate (THF) in the folic acid cycle. Folic acid/folate is activated to 5-methyl THF by the activity of methylenetetrahydrofolate reductase (MTHFR) [3].

B vitamins include eight water-soluble vitamins that consist of thiamine (B_1_), riboflavin (B_2_), niacin (B_3_), pantothenic acid (B_5_), pyridoxine (B_6_), biotin (B_7_), folic acid (B_9_), and cobalamin (B_12_) [1]. B vitamins are eliminated through the urine and must be replenished daily through dairy products, leafy green vegetables, or food of animal origin [1]. They are cofactors for cellular pathways that support physiological function [2]. Specifically, vitamins B_6_, folic acid, and B_12_ are involved in the homocysteine metabolic pathway, a pathway shown to be associated with thrombotic risk reduction (Figure 1) [1,2].

Homocysteine (Hcy), derived from the essential amino acid methionine, is known to play a role in cellular homeostasis. High concentrations of homocysteine in plasma may induce oxidative damage to endothelial cells, which consequently causes dysfunction of the anticoagulation system, leading to thrombotic events [4]. Elevated plasma homocysteine levels, or hyperhomocysteinemia, have been found to be linked to cardiovascular disease and venous thrombotic events [5,6,7,8,9]. Several studies have suggested that hyperhomocysteinemia (>15 µmol/L) may negatively affect the cardiovascular system and lead to ischemic stroke, coronary artery disease, and deep vein thrombosis [10]. In particular, when homocysteine levels went above 30 µmol/L (severe/moderate hyperhomocysteinemia), a positive correlation between homocysteine levels and thromboembolic risk was relatively frequently demonstrated [10,11]. However, when homocysteine was modestly elevated (15–30 µmol/L), it had mixed cardiovascular effects. Therefore, it is challenging to demonstrate that hyperhomocysteinemia is an independent risk factor for thromboembolism [10].

Additionally, hyperhomocysteinemia was noted to be associated with vitamin B deficiency. However, whether B vitamin supplementation may improve clinical outcomes of arterial or venous thromboembolisms remains uncertain. The United States implemented regulations for folic acid fortification beginning in 1996. This policy change had the potential to impact the findings of studies examining folic acid and its associated clinical effects. Our study aims to review the randomized controlled trials (RCTs) published since 1996, and to assess the effects of B vitamin supplementation—mainly vitamin B_6_, folic acid, and vitamin B_12_—on the risk of arterial and venous thromboembolic events, as well as other relevant clinical outcomes.

## 2. Materials and Methods

### 2.1. Search Strategy and Study Selection

**Figure 2:**
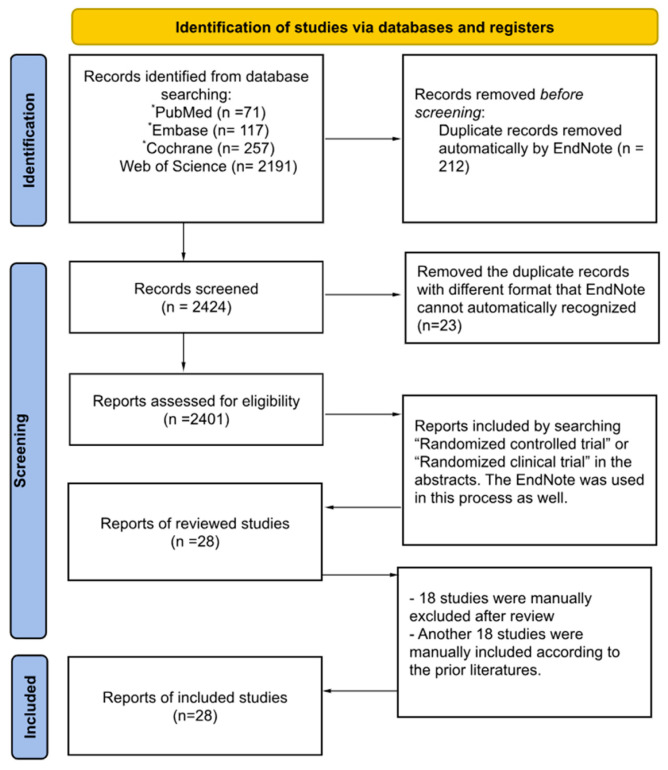
Preferred Reporting Items for Systematic Reviews and Meta-Analyses (PRISMA) flow diagram of study selection based on inclusion and exclusion criteria [12]. * The article types, such as trial and randomized clinical trial, can be selected while searching PubMed, Embase and Cochrane; however, this option is not available in Web of Science.

A literature search was conducted using four databases (PubMed, Embase, Web of Science, and Cochrane) to identify all RCTs published between January 1996 and February 2025 using predefined search terms: (vitamin B OR folate OR folic acid OR B vitamins) AND (homocysteine OR homocysteinemia OR hyperhomocysteinemia) AND (thrombosis OR thrombotic OR cardiovascular event OR stroke OR cardiovascular accident OR thromboembolism), see Figure 2.

### 2.2. Eligibility Criteria

Trials were eligible if they fulfilled the following requirements:

1. Population: adult patients (greater or equal to 18 years of age).
2. Intervention: oral, enteral, or parenteral folic acid (Vitamin B_9_) and/or cobalamin (or vitamin B_12_) and/or pyridoxine (vitamin B_6_) with or without standard therapy.
3. Outcomes: Incidence of any thrombotic events, including (but not limited to) myocardial infarction (MI), stroke or transient ischemic attack (TIA), cardiovascular accident (CVA), deep vein thrombosis (DVT), pulmonary embolism (PE). Trials reporting only biochemical outcomes or surrogate markers were excluded.

### 2.3. Eligibility Review and Data Abstraction 

The primary literature screening was performed using the keywords listed above in the four databases. All of the authors were independently assigned to review the literature after the primary screening. Details about the vitamin B supplementation regimen, study methods, homocysteine levels, and clinical outcomes were extracted. 

### 2.4. Qualitative Analysis

For each of the included RCTs, two reviewers independently evaluated the methodological quality, risk of bias, and synthesis of the results. Disagreements between reviewers were resolved through discussion or third-party adjudication. The list of final RCTs was cross-verified against published meta-analyses to ensure completeness and prevent omissions. No quantitative analysis was performed due to the heterogeneity of the studies.

## 3. Results

Twenty-eight randomized controlled trials (RCTs) met our inclusion criteria (Table 1 and Table 2). Twenty-six of these studies mainly focused on the outcomes of arterial thrombosis and two of them mainly focused on venous thrombotic events. Various vitamin B supplementation strategies have been used, with dosing regimens varying from folic acid alone to combinations of folic acid, vitamin B_6_, and vitamin B_12_. See Table 1 for a brief summary, which includes the study design and B vitamins regimen, and Table 2 for a summary of the clinical outcomes. More details of Table 1 can be found in the Supplementary Table S1. 

### 3.1. Summary of Trials on Arterial Thrombosis Events

Among the eight trials [13,15,16,18,32,33,37,40,41] that investigated single-ingredient folic acid supplementation as the intervention, only three studies [13,15,16] demonstrated a significant reduction in the risk of first ischemic stroke. Folic acid was not found to have significant benefits in preventing MI, stroke (including ischemic stroke, hemorrhagic stroke, and cerebrovascular disease), or mortality from the other five trials [28,32,33,35,37,41] (Refs [33,41] from the same trial). Similarly, supplementation with combination folic acid and vitamin B_12_ and vitamin B_6_ alone did not yield a significant reduction in stroke, MI, or mortality as either a primary or secondary preventive measure [14,25].

Studies examining the combined supplementation of folic acid, vitamin B_6_, and vitamin B_12_ have produced mixed results [19,20,22,24,34]. While the majority of studies found no significant differences in clinical outcomes among populations receiving this combination, research by Galan et al. and the HOPE trial reported a significantly positive preventive effect on stroke [8,22]. However, Galan et al.’s study also identified a significantly increased risk of all-cause mortality in the B vitamin supplementation group compared to the placebo group (5.8% vs. 3.6%, *p* = 0.02) [22]. Likewise, the NORVIT trial found that B vitamin supplementation significantly increased the risk of non-fatal MI [31].

Six of the twenty-six studies were based on the China Stroke Primary Prevention Trial (CSPPT) with different analysis of the effects of enalapril with or without folic acid supplementation in adults with hypertension and without history of arterial thrombotic events [17]. In the CSPPT, folic acid supplementation significantly reduced the occurrence of first stroke, first ischemic stroke, and composite cardiovascular events. In particular, the risk of first stroke was significantly reduced by 73% in a subgroup with a low platelet count (<210 × 10^9^/L) and high total homocysteine (tHcy) (≥15 μmol/L) [15]. The effect of folic acid intervention also significantly reduced stroke risk in patients with the CC/CT MTHFR genotype [16] and in male patients with elevated serum calcium levels (albumin-corrected serum calcium ≥2.43 mmol/L) [13].

In the VISP trial, which examined a post-ischemic stroke population, no significant difference in stroke risk was observed between patients receiving high-dose B vitamin therapy (vitamin B_6_: 25 mg, vitamin B_12_: 0.4 mg, and folic acid: 2.5 mg) and those receiving low-dose therapy (vitamin B_6_: 200 μg, vitamin B_12_: 6 μg, and folic acid: 20 μg) [36]. In a subgroup analysis targeting patients most likely to benefit from vitamin therapy, a significant reduction in the risk of ischemic stroke, coronary artery disease, and death was observed in the high-dose group [34]. This analysis excluded patients with low or very high baseline B_12_ levels (<250 and >637 pmol/L, representing the 25th and 95th percentiles) and those with a low glomerular filtration rate to minimize potential confounding from B12 malabsorption or additional B12 supplementation [34]. Furthermore, patients with severe renal failure requiring dialysis were excluded, as they typically exhibit markedly elevated homocysteine levels that are known to be unresponsive to vitamin supplementation. Interestingly, in the post hoc analysis of the population receiving antiplatelet therapy, VISP trial showed higher ischemic stroke risk for patients supplemented with high-dose B vitamins therapy, compared with those on low-dose therapy. No significant difference in ischemic stroke risk was observed between the high-dose and low-dose groups among those not receiving antiplatelets [19].

In the VITATOPS trial, which included patients with a history of stroke or TIA, supplementation with folic acid, vitamin B_6_, and vitamin B_12_ did not result in a significant reduction in non-fatal stroke, non-fatal MI, or vascular death compared to placebo [20,24]. For patients who were not receiving antiplatelet therapy at baseline, B vitamin combination supplementation was shown to significantly decrease the risk of recurrent ischemic stroke and cardiovascular-related death. Consistent with findings from the VISP trial, this benefit was not observed in patients who were using antiplatelets at baseline [36].

### 3.2. Summary of Trials on Venous Thrombotic Events

For studies using folic acid and vitamin B_12_ combination supplementation, Shu et al. found that the recurrence rate of lower limb DVT of the treatment group was 4.4%, which was significantly lower than that (28.9%) of the non-treatment group in patients with cerebral infarction with DVT and baseline homocysteine levels around 30 µmol/L [38]. The risk-lowering effect of PE was not found in the SEARCH trial in the population with a history of MI [25].

A combination of folic acid, vitamin B_6_, and vitamin B_12_ did not demonstrate a significant reduction in venous thromboembolism (VTE) events as non-primary outcomes of the studies in the HOPE-2 and VITRO trials and a study conducted by Heinz et al. [8,26,39]. Notably, Kotwal et al. reported a lower number of incidences of DVT and PE in soldiers stationed at high altitudes following supplementation with folic acid, vitamin B_6_, and vitamin B_12_ [18].

### 3.3. Summary of Homocysteine-Lowering Effects

Overall, the homocysteine-lowering effects of B vitamin supplementation were observed across studies [22,24,25,31,38,39] (see Table 3). Most studies either demonstrated a significant reduction in thrombotic events with decreasing homocysteine levels or found no significant difference between the placebo and intervention groups [14,17,24,28,31,36,39]. Patients’ serum folic acid and vitamin B_12_ levels were also found to increase with vitamin B supplementation. Homocysteine levels decreased with vitamin B supplementation and exhibited a negative correlation with folic acid and vitamin B12 levels. [35]. However, in a study involving end-stage renal disease (ESRD) patients on dialysis, a reverse trend was observed between homocysteine levels and clinical events [35].

## 4. Discussion

### 4.1. Effects of Vitamin B Supplements on Arterial Thrombotic Events

The debated role of vitamin B supplements in reducing stroke risk by lowering homocysteine levels has been highlighted in previous studies [15,24,34]. One of the studies from the CSPPT [17] demonstrated that a combination of antihypertensive medication and folic acid could reduce the risk of stroke by 21%, compared to antihypertensive medication alone. A sub analysis showed that the patients with low platelet count and high tHcy had the highest risk of first stroke, and this risk can be reduced by 73% with folic acid supplement [15]. On the other hand, the VISP trial [36] and VITATOPS [24] showed no significant difference in reducing the primary outcome of recurrent stroke between the high-dose folic acid, vitamin B_6_ and B_12_ (2.5 mg, 25 mg, and 0.4 mg) group and the low-dose (folic acid 20 μg, vitamin B_6_ 200 μg, and vitamin B_12_ 6 μg) group. The trials also showed no differences in reducing the composite outcomes of recurrent stroke or TIA when comparing the folic acid groups with the placebo group, respectively [24,36]. These conflicting results suggest the clinical effects of vitamin B supplement on stroke risk reduction remain indeterminate.

Furthermore, the effects of vitamin B supplements on arterial cardiovascular events may be modified by antiplatelet therapy, which is the standard of care to prevent arterial cardiovascular events. The post hoc analyses of the VISP trial showed that high-dose folic acid with vitamin B_6_ and B_12_ increased the risk of recurrent stroke in patients receiving the concurrent antiplatelet therapy compared to a low dose [19]. However, among those who were not on antiplatelet therapy, a trend of decreased risk was seen with high doses of folic acid. Similar trends regarding the role of B vitamins in risk reduction for stroke, MI, or cardiovascular death were reported in the post hoc analyses of the VITATOPS trial [20]. The aforementioned evidence demonstrated that antiplatelet therapy can be regarded as a modifier of the antithrombotic effects of B vitamins [19]. The mechanism of interaction between antiplatelet therapy and homocysteine-reducing therapy has yet to be determined. A possible explanation is that aspirin, as well as B vitamins, which are involved in homocysteine-lowering therapy, may interfere with each other in the prevention of arterial thrombosis [19,42]. Aspirin inhibits the production of thromboxane A₂ in platelets, reducing platelet activation and aggregation, thereby preventing arterial thrombosis [43]. Homocysteine, on the other hand, can induce oxidative stress, enhance prothrombotic platelet function, and activate platelets, potentially leading to thrombosis [44]. The presence of antiplatelet therapy may attenuate the platelet-modulating effects of homocysteine-lowering therapy, potentially compromising each other’s efficacy in thrombosis prevention.

These findings suggest that antiplatelet-naive patients could be a targeted population for B vitamin treatment aimed at the prevention of stroke and other cardiovascular thrombotic events. As the current results are from post hoc analyses, more prospective studies are warranted to investigate the correlation between antiplatelet therapy, B vitamins, and the risks of arterial thrombotic events.

Interestingly, trials investigating the combined effects of folic acid, vitamin B6, and vitamin B12 have yielded mixed results, showing both significant positive and negative outcomes. In the three studies reporting negative outcomes [19,22,31], these negative findings were observed as non-primary endpoints in populations with a history of myocardial infarction (MI) or stroke. These populations were generally sicker at baseline compared to those using B vitamins for primary prevention. Since the negative outcomes were measured as non-primary endpoints, baseline comorbidities in these patients may have introduced confounding effects, warranting cautious interpretation of the results.

### 4.2. Effects of Vitamin B Supplements on Venous Thrombotic Events (VTEs)

Different members of the B vitamin family have different correlations with venous thrombosis [45]. The previous studies confirm no correlation between thiamine (B_1_), riboflavin (B_2_), niacin (B_3_) and pantothenic acid (B_5_) with venous thrombosis [45]. A meta-analysis result suggested the reduced level of folic acid and vitamin B_12_ may be independent risk factors for venous thrombosis, which was regardless of the baseline homocysteine level [45]. A case–controlled study by Cattaneo et al. also found the correlation between low vitamin B_6_ levels and the risk of DVT, independent of fasting homocysteine levels [46]. Additionally, significantly reduced vitamin B_6_ levels were detected among patients with first DVT, compared to healthy volunteers [46]. These findings suggested that low levels of vitamin B_6_ may also be a risk factor for venous thrombosis. Putting them together, we suspected that supplementing folic acid, vitamin B_12_ or Vitamin B_6_ may reduce the risk of VTE, which may or may not be independent of the homocysteine-lowering pathway.

However, when we assessed the effect of B vitamin supplementation on the risk of a reduction in VTEs through the homocysteine-lowering mechanism, conflicting results were observed. In the VITRO trial, adult patients with a first confirmed DVT or PE with a homocysteine level above the 75th percentile compared to the normal value were randomized to the group with daily supplementation of folic acid, vitamin B_6_, and vitamin B_12_ or to a placebo. The number of recurrent VTE was 12.2% in the B vitamin group vs. 14.4% in the placebo group without a significant difference [39]. This suggested that homocysteine lowering by B vitamins did not prevent any recurrent VTE [39]. Furthermore, in the hyperhomocysteinemic group (mean baseline tHcy between 15.1 and 15.9) of the VITRO trial, B vitamin treatment seemed to be associated with higher cumulative incidence of recurrent thrombosis than the placebo. On the contrary, in another RCT enrolling 90 patients with history of homocysteine cerebral infarction and DVT, the recurrence rate of thrombosis was significantly reduced with folic acid and vitamin B_12_ supplementation [47]. It was also observed that homocysteine level was negatively correlated with folic acid and vitamin B_12_ [47]. These conflicting findings suggested that the clinical effects of B vitamins on VTE risk reduction still remains undetermined. The homocysteine-lowering pathway might not be the only mechanism involving clinical effects of B vitamin supplementation on thrombosis prevention. Further studies are needed to clarify these suspicions.

### 4.3. Effects of Vitamin B Supplements on Other Vascular Outcomes

Vascular endothelial function was reportedly improved by supplementation of B vitamins. For example, Chambers et al. demonstrated that oral folic acid and vitamin B_12_ supplementations improved vascular endothelial function in patients with coronary artery disease. The mechanism is thought to occur via reducing homocysteine levels in the body [48]. Similarly, Menzel et al. also demonstrated that B vitamins could reduce the deterioration of endothelial function, blood pressure and tHcy levels [49]. Zamani et al. published a systematic review in 2023 which addressed folic acid’s positive effect on endothelial function [50]. The possible mechanism of the folic acid’s effect could be associated with elevation in flow-mediated dilation percentage levels [50].

Other vascular outcomes were also reviewed. Low vitamin B_6_ and folic acid levels, along with elevated homocysteine levels, are independent risk factors for retinal vein occlusion [51]. Meng et al. published a study in 2018 and demonstrated that the prevalence of retinal atherosclerosis (RA) was 77.6% in patients with hypertension and diabetes, and folic acid supplementation was associated with reduced RA in female patients with hyperhomocysteinemia [52]. In addition, Hodis et al. showed that high-dose vitamin B supplementation reduced the progression of early-stage subclinical atherosclerosis (carotid artery intima–media thickness) in well-nourished individuals at low risk of cardiovascular disease with a fasting homocysteine level of >9.1 µmol/L [53]. Finally, vitamin B supplementation could improve arterial endothelial function in vegetarians with subnormal vitamin B_12_ levels [54]. Therefore, since endothelial dysfunction is a risk factor for cardiovascular disease, including hypertension and atherosclerosis, B vitamin supplements may have protective effects against these cardiovascular diseases.

### 4.4. Effects of Vitamin B Supplements and tHcy Lowering

It has been shown that vitamins B_6_, folic acid, and B_12_ are involved in the homocysteine metabolic pathway [1,2]. The clinical effect of vitamin B was confirmed in the CSPPT, in which folic acid supplementation was shown to decrease the tHcy level and the degree of the tHcy-level reduction was affected by sex, MTHFR C677T genotypes, baseline folate, tHcy, estimated glomerular filtration rate levels, and smoking status [55]. Homocysteine-lowering response was eliminated by genotype when plasma folate levels reached ≈15 ng/mL or higher [55]. This suggests that the homocysteine-lowering pathways may be involved in the clinical role of B vitamin supplementation.

### 4.5. Potential Cofounders on Clinical Trial Outcomes

#### 4.5.1. Dietary Fortification and Nutritional Deficiencies

Mandatory fortification of grains in the US may be one of the reasons that caused discrepancies regarding reducing stroke risk between trials from North America and trials from the rest of the world. Since 1996, when fortification became mandatory, the mean tHcy level of the population was lowered from 10.1 to 9.4 μmol/L (*p* < 0.001) and the prevalence of a high homocysteine level (>13 µmol/L) went down from 18.7% to 9.8% (*p* < 0.001) [56]. Therefore, fortification in North America may be presented as a confounding factor when determining the effects of B vitamins on reducing the risks of thrombotic events, in which homocysteine-lowering pathways may be involved [57,58]. On the other hand, most of Europe and China do not mandate fortification. A meta-analysis showed a modest reduction in future strokes with the use of folic acid in countries with non-mandatory fortification (RR, 0.85; 95% CI, 0.77 to 0.95) [59]. This suggested that the benefits of B vitamin supplementation for prevention of thromboembolism may only apply in populations with very-high-baseline homocysteine due to lack of mandatory fortification.

#### 4.5.2. Concurrent Medication Treatment

Some medications could decrease the absorption of vitamin B_6_, B_9_ and B_12_ or trigger hyperhomocysteinemia [60]. For example, antiepileptics and sulfasalazine can reduce the absorption of folic acid. Isoniazid, cycloserine, penicillamine, hydralazine, levodopa, and some anticonvulsants could affect vitamin B_6_ absorption. Proton pump inhibitors, H_2_ receptor antagonists, colchicine, and metformin could lead to vitamin B_12_ malabsorption. Medications such as methotrexate, niacin, and cholestyramine can also cause hyperhomocysteinemia [60]. There is heterogeneity in terms of home medication reporting from the clinical trials evaluated in this review. Most studies only listed general classes of home medications without specifically collecting data on medications with potential interactions with B vitamins. Therefore, we speculated that drug–drug interaction may play a role in the conflicting results.

#### 4.5.3. Genetic Mutations

Mutation in the MTHFR gene is associated with reduced enzymatic efficiency of MTHFR and increased homocysteine levels. One of the common genetic variants, MTHFR 677C → T, has been identified as one of the causes of hyperhomocysteinemia [61]. As the genotype C677T (heterozygous) was associated with a mildly increased homocysteine level, the homozygous T677T polymorphism elevated the homocysteine level by 25% compared to the CC genotype (noncarriers) [61,62,63]. The homozygous TT genotype has been found more frequently in the Chinese population than in other populations. This genotype is associated with a 13% increase in the risk of any type of stroke (adjusted odds ratio 1.13, 95% CI 1.09–1.17) when compared to noncarriers [63]. MTHFR polymorphism of C677T varies based on geography and ethnicity. Approximately 25% of the world’s population are MTHFR 677T carriers. The MTHFR polymorphism is most prevalent in Hispanic people (47%), East Asian people (30%) and Europeans (36%). Africans are the least affected by MTHFR polymorphism [64]. This genetic distribution may lead to a higher risk of hyperhomocysteinemia and improve the efficacy of folic acid supplementation among the Chinese population for the prevention of stroke. One of the CSPPT post hoc analyses focused on the MTHFR mutation subgroup and identified that folic acid benefited patients with the TT genotype and a low platelet count the most, with a risk reduction of 66% (HR 95%CI, 0.15–0.81) [65]. Since genetic testing for MTHFR polymorphism is becoming increasingly accessible for clinicians, with a reasonable laboratory turnaround time (6-10 days) and in some cases may be commercially available in the form of home test kits, knowing the MTHFR variants’ data may help target the population that will benefit the most from B vitamin supplementation.

#### 4.5.4. 5-Methyltetrahydrofolate

There was only one study in our review that used 5-Methyltetrahydrofolate (5-Methyl-THF) instead of folic acid as the intervention [22]. Natural sources of 5-methyl-THF may provide more advantages than synthetic folic acid among specific populations [66]. As the activation of folic acid to 5-methyl-THF is based on the activity of MTHFR, people carrying MTHFR mutated genes may benefit more from taking 5-methyl-THF instead of folic acid due to its improved bioavailability. Additionally, instead of synthetic folic acid, 5-methyl-THF is associated with reduced inflammation, prolonged survival, and decreased all-cause mortality in ESRD patients [67,68]. Moreover, taking 5-methyl-THF may help prevent the masking of pernicious anemia caused by vitamin B_12_ deficiency, reduce interactions with drugs that inhibit dihydrofolate reductase, and reduce the accumulation of unmetabolized folic acid and its potential side effects [66,69]. However, Galan et al did not show a significant benefit of B vitamins, including 5-methyl-THF, on decreasing the risk of stroke [22].

#### 4.5.5. Safety of Vitamin B Supplement

Though B vitamins are water-soluble vitamins with a wide therapeutic index, over-supplementing B vitamins could also lead to unwanted clinical outcomes. It was observed in a previous study that within the folic acid dose range of 0–1.2 mg/day, the homocysteine level was steadily lowered by an increase in folic acid [70]. However, the lowering effect plateaued in a dose range of 1.2–1.6 mg, which indicated the ceiling effect of folic acid. Based on another meta-analysis [6], a 1 mg/day dose of folic acid produced the maximum reduction in the homocysteine level, with no additional decrease observed at higher doses (up to 5 mg/day). Folic acid is currently added to enriched white flour, cornmeal, and pasta in countries with mandatory folic fortification. In some cases, folic supplementation could mask pernicious anemia caused by vitamin B12 deficiency. Additionally, the human body’s capacity to incorporate folic acid into the one-carbon metabolic pathway is limited. Over-supplementation of folic acid could lead to unmetabolized synthetic folic acid, which has emerged as a risk factor for gestational diabetes in pregnancy [71]. Concerns also have been raised about its potential unwanted effect in regard to cancer, depression, and cognitive impairment [72]. The European Scientific Committee on Food has thus established a tolerable upper intake limit of folic acid of 1mg/day [73]. A similar upper limitation was also set by the US authority [74]. Over-supplementation of vitamin B_6_ could also lead to peripheral neuropathy past its recommended upper limit of 100 mg/day. Furthermore, in 2020, Flores-Guerrero et al. performed a prospective population-based cohort study which demonstrated that higher levels of plasma concentrations of vitamin B_12_ were associated with increased risk of all-cause mortality after adjusting for age, sex, renal function, and other clinical and laboratory variables. Caution should be exercised when considering vitamin B_12_ supplementation in the absence of vitamin B_12_ deficiency [75]. The DIVINe trial (Diabetic Intervention with Vitamins to Improve Nephropathy) also found that vitamin B_12_ may decrease the glomerular filtration rate and increase vascular events for participants with impaired renal function [23]. In conclusion, the possible harmful effects of over-supplementation in the general population and supplementation in the renal impairment group can mitigate the potential beneficial effects and should be avoided.

### 4.6. Limitations and Further Research

Our review is limited by narrative nature that no quantitative analysis was performed due to the heterogeneity of the trial designs. Our literature search included RCTs published in the last 29 years (January 1996 to February 2025) from various countries. We noticed that comparing outcome measures is no longer meaningful or relevant when looking at data from older studies or from different countries. Also, when we looked into the subgroup analysis for age, genders, races, smoking and some baseline medical conditions such as diabetes and cardiovascular disease, we found that small trials cannot provide enough power to show the statistical difference. But large trials showed some uncomparable results. For example, the VISP trial showed no effects of B vitamin treatment within various participant subgroups [36]. Jamison et al. observed similar all-cause mortality between the treatment and placebo group in the subgroups examined in their study [29]. But the CSPPT investigators found that folic acid therapy had different effects on reducing stroke risks for the participants with different genotypes of MTHFR [13,15,16,17]. It became difficult to perform subgroup analyses among those trials. Moreover, our review relies on clinical trials, each of which may have its own limitations. These raised uncertainty when we tried to reach some conclusions. Therefore, we suggested that well-designed clinical trials are needed, which should consider patients’ baseline social and demographic information, such as their baseline vitamin B and homocysteine level, smoking and alcohol use, and current medication use. Underlying conditions such as hypercholesterolemia, obesity, and genetic mutations also need to be considered for a more homogenous cohort that may yield definitive results. A meta-analysis focusing on subgroups might help us to understand the complexities of the clinical effects of B vitamins.

## 5. Conclusions

This review investigated the effect of B vitamin supplementation on thrombotic risk by analyzing clinical trials published in the last 29 years. Limited studies were found, with conflicting results regarding thrombotic risk reduction in association with supplementation of B vitamins. The homocysteine-lowering pathway may not be the only mechanism involved in the clinical role of B vitamins. Thus, more clinical and translational studies are needed to determine a clearer correlation between B vitamin supplementation and the risk of thrombosis.

## Figures and Tables

**Table 1:** Summary of RCT study designs on the effect of vitamin b supplements on the risk of thrombotic events (January 1996–February 2025).

Study	Sample Size and Population	Intervention and Comparison	Duration
**Trials on Arterial Thrombotic Events**			
Wu et al. CSPPT 2021 [13]	20,424 hypertensive adults without a history of stroke or MI	Enalapril 10 mg and folic acid 0.8 mg (single pill) daily vs. Enalapril 10 mg daily alone	4.5 years
Oliari Araghi et al., B-PROOF trial extended follow-up 2021 [14]	1298 patients with aged ≥65 with an elevated Hcy level (12–50 µmol/L)	Folic acid (400 µg daily) and vitamin B_12_ (500 µg daily) vs. placebo	5–7 years
Kong et al. Post-hoc analysis of CSPPT 2018 [15]	10,789 hypertensive adults without a history of stroke or MI	Enalapril 10 mg–Folic acid 0.8 mg (single pill) daily vs. Enalapril 10 mg daily alone	4.5 years (median)
Zhao et al. Post-hoc analysis of CSPPT 2017 [16]	20,424 hypertensive adults without a history of stroke or MI	Enalapril 10 mg–folic acid 0.8 mg (single pill) daily vs. Enalapril 10 mg daily alone	4.5 years (median)
Huo et al. The CSPPT trial 2015 [17]	20,424 hypertensive adults without a history of stroke or MI	Enalapril 10 mg–folic acid 0.8 mg (single pill) daily vs. Enalapril 10 mg daily alone	4.5 years (median)
Kotwal et al., 2015 [18]	6000 Armed Forces personnel in the high-altitude area	Vitamin B_12_ 1000 μg, B_6_ 3 mg and folic 5 mg daily vs. no treatment	2 years
Arshi et al. Post hoc analysis of VISP 2015 [19]	3680 patients with non-disabling post-ischemic stroke	High dose group (vitamin B_6_ 25 mg, vitamin B_12_ 0.4 mg, and folic acid 2.5 mg daily) vs. Low dose group (vitamin B_6_ 200 μg, vitamin B_12_ 6 μg, and folic acid 20 μg daily)	2 years
Hankey et al., VITATOPS post-hoc analysis 2012 [20]	8164 patients with recent stroke or TIA (within the past 7 months)	Folic acid 2 mg, vitamin B_6_ 25 mg, and vitamin B_12_ 0.5 mg daily vs. placebo	3.4 years
Bostom et al., 2011 [21]	4110 patients with stable kidney transplant recipients	High dose: folic acid 5.0 mg, vitamin B_6_ 50 mg, and vitamin B_12_ 1.0 mg daily; Low dose: vitamin B_6_ 1.4 mg and vitamin B_12_ 2.0 µg daily	4 years
Galan et al., 2010 [22]	2501 patients with history of MI, UA or ischemic stroke	5-methyltetrahydrofolate (5-methyl-THF) 560 μg, vitamin B_6_ 3 mg, and vitamin B_12_ 20 μg daily vs. placebo	4.7 years
House et al., 2010 [23]	238 patients with diabetes and diagnosed diabetic nephropathy	Folic acid 2.5 mg, vitamin B_6_ 25 mg, and vitamin B_12_ 1 mg daily vs. placebo	2.7 years
VITATOPS trial 2010 [24]	8164 patients with recent stroke or TIA (within the past 7 months)	Folic acid 2 mg, vitamin B_6_ 25 mg, and vitamin B_12_ 0.5 mg daily vs. placebo	3.4 years
SEARCH trial 2010 [25]	12,064 survivors of MI	Folic acid 2 mg and vitamin B_12_ 1 mg daily vs. placebo	6.7 years
Heinz et al., 2010 [26]	650 patients with ESRD	Active treatment: folic acid 5 mg, vitamin B_12_ 50 μg, and vitamin B_6_ 20 mg given 3 times a week; Placebo: folic acid 0.2 mg, vitamin B_12_ 4 μg, and vitamin B_6_ 1.0 mg, given 3 times a week.	2.1 years [median]
Albert et al., 2008 [27]	5442 female US health professionals with either a history of CVD or ≥three coronary risk factors	Folic acid 2.5 mg, vitamin B_6_ 50 mg, and vitamin B_12_ 1 mg daily vs. placebo	7.3 years
Ebbing et al. WENBIT trial 2008 [28]	3096 patients undergoing coronary angiography	Four groups: folic acid 0.8 plus vitamin B_12_ 0.4 mg plus vitamin B_6_ 40 mg daily; folic acid plus vitamin B_12_ daily; vitamin B_6_ daily alone; and placebo.	3.2 years [median]
Jamison et al., 2007 [29]	2056 patients with advanced CKD (eCrCl 30 mL/min) or ESRD, and high Hcy levels (≥15 µmol/L).	Folic acid 40 mg, vitamin B_6_ 100 mg, and vitamin B_12_ 2 mg daily vs. placebo	3.2 years [median]
HOPE 2 trial 2006 [30]	5522 patients with vascular disease or diabetes	Folic acid 2.5 mg, vitamin B_6_ 50 mg, and vitamin B_12_ 1 mg daily vs. placebo	5 years
Bønaa et al. NORVIT trial 2006 [31]	3749 patients who had an acute MI within 7 days before randomization	Four groups: G1: folic acid 0.8 mg, vitamin B_12_ 0.4 mg, and vitamin B_6_ 40 mg daily; G2: folic acid 0.8 mg and vitamin B_12_ 0.4 mg daily; G3: vitamin B_6_ 40 mg daily; vs. G4: placebo	3.3 years [median]
Zoungas et al., ASFAST trial 2006 [32]	315 patients with ESRD	folic acid 15 mg daily vs. placebo	3.6 years [median]
Liem et al., 2005 [33]	593 patients with stable CAD	folic acid 0.5 mg vs. standard care	3.5 years
Spence et al., VISP trial-subgroup analysis 2005 [34]	2155 patients with non disabling post-ischemic stroke, baseline vitamin B_12_ in between the 25th percentile and the 95th percentile, GFR ≥ the 10th percentile	High-dose group (vitamin B_6_ 25 mg, vitamin B_12_ 0.4 mg, and folic acid 2.5 mg daily) vs. low-dose group (vitamin B_6_ 200 μg, vitamin B_12_ 6 μg, and folic acid 20 μg daily)	2 years
Wrone 2004 [35]	510 patients with ESRD on dialysis	1, 5, or 15 mg of folic acid contained in a renal multivitamin	2 years [median]
Toole et al., VISP trial 2004 [36]	3680 non-disabling post-ischemic stroke	High-dose group (vitamin B_6_ 25 mg, vitamin B_12_ 0.4 mg, and folic acid 2.5 mg daily) vs. low-dose group (vitamin B_6_ 200 μg, vitamin B_12_ 6 μg, and folic acid 20 μg daily)	2 years
Liem, et al., 2004 [37]	283 patients with a total cholesterol > 251 mg/dL	Folic acid 5 mg plus fluvastatin 40 mg daily vs. fluvastatin 40 mg daily	1 year
**Trials on Venous Thrombotic Events**			
Shu et al., 2017 [38]	90 patients with homocysteine cerebral infarction	Folic acid 5 mg and vitamin B_12_ 0.25 mg daily vs. no treatment	3 months
den Heijer et al. The VITRO trial 2007 [39]	701 patients between 20 and 80 years of age with a first objectively confirmed DVT/PE	Folic acid 5 mg, cyanocobalamin 0.4 mg, and pyridoxine 50 mg daily vs. placebo	2.5 years

Hcy: homocysteine; CVD: cardiovascular disease; CV: cardiovascular; MI: myocardial infarction; eGFR: estimated glomerular filtration rate; ESRD: end-stage renal disease; CKD: chronic kidney disease; eCrCl: estimated creatinine clearance; PAI 1: plasminogen activator inhibitor-1; NO: nitric oxide; CAD: coronary artery disease; CABG: coronary artery bypass graft; ACS: acute coronary syndrome; SVT: supraventricular tachycardia; HF: heart failure; DVT: deep vein thrombosis; PE: pulmonary embolism; VTE: venous thrombotic events; FA: folic acid; MTHFR: methylenetetrahydrofolate reductase; CC/CT genotype: wild type (C), mutated type (T); TT-homozygous genotype, CT-heterozygous genotype, CC-noncarrier; INR, PT, APTT: international normalized ratio, prothrombin time, activated partial thromboplastin time; HR: hazard ratio; CI: confidence interval; RR: risk ratio; py: patient-years.

**Table 2:** Summary of RCT study outcomes on the effect of vitamin B supplements on the risk of arterial and venous thrombotic events (January 1996–February 2025).

Intervention	Arterial Thrombotic Events						Venous Thrombotic Events			Safety
	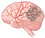 First Ischemic Stroke	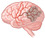 Recurrent Ischemic Stroke	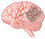 Undefined Stroke *	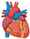 MI	Death from CV Cause	Death From Any Cause	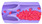 DVT (Only)	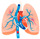 PE (Only)	VTE (DVT/PE)	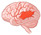 Hemorrhagic Stroke
**FA**	Kong (pts w/low plt) [15], Zhao [16], Wu [13]		Ebbing [28], Liem (2005) [33], Wrone [35], Liem (2004) [37], Zoungas [32]	Ebbing [28], Wrone [35], Liem 2004 [37], Zoungas [32]	Zoungas [32]	Ebbing [28], Liem (2005) [33], Wrone [35]				Huo [17]
**B_6_**			Ebbing [28]	Ebbing [28]		Ebbing [28]				
**FA+B_12_**			Oliari Araghi [14], SEARCH [25]	Oliari Araghi [14], SEARCH [25]	SEARCH [25]	SEARCH [25]	Shu [38]	SEARCH [25]		
**FA+B_6_+B_12_**		Arshi (pts w/antiplatelets) [19]	Galan [22], HOPE2 [8]	NORVIT (non-fatal) [31]	VITATOPS [24], Hankey (w/o antiplatelets) [20]	Galan [22]	Kotwal [18]	Heinz [26]	HOPE2 [8], VITRO [39], Kotwal [18]	Albert [27]

		Hankey (w/o antiplatelets) [20]								
				Hankey [20], Bostom [35], House [23], VITATOPS [24], Heinz [26], Albert [27], Jamison [29], Kotwal [18]	Hankey (w/antiplatelets) [20], Galan [22], Bostom [21], Heinz [26], Albert [27], HOPE2 [8], Lonn [30]					
						Bostom [21], House [23], VITATOPS [24], Heinz [26], Albert [27], Jamison [29], HOPE2 [8], Toole [36], NORVIT [31]				
		Spence [34], Hankey (w/antiplatelets) [20], VITATOPS [24], Toole [36]	Bostom [21], House [23], Heinz [26], Albert [27], Jamison [29], NORVIT [31], Kotwal [18]							

Note: * All patients with this outcome were included; outcomes included ischemic stroke, hemorrhagic stroke, and cerebrovascular disease. 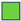 Results showed significant decreased risk; 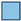 results showed no significant changes; *p*-values/95% confidence interval not reported; 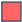 results showed significant increased risk; 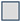 results not reported. FA: folic acid group; B_6_: vitamin B_6_ group; B_12_: vitamin B_12_ group. DVT: deep venous thrombosis; PE: pulmonary embolism; VTE: venous thromboembolism; CV: cardiovascular; MI: myocardial infarction; pts: patients; w/: with; w/o: without; plt: platelet.

**Table 3:** Summary of RCT study baseline characteristics and changes in serum homocysteine, folic acid, and vitamin B12 levels (January 1996–February 2025).

Author, Year	Country	Mandatory Folic Fortification At the Time of the Study	Age (years)	History of Thrombotic Event	History of CKD	Homocysteine (µmol/L) Baseline/after Treatment	Folic Acid (ng/mL) Baseline/after Treatment	Vitamin B_12_ (pg/mL) Baseline/after Treatment
**Trials on Arterial Thrombotic Events**								
Huo et al. (CSPPT, 2015) [17]	China	No	60.0 ± 7.5	No	No	12.5 (10.5–15.5)/N/A	8.1 (5.6–10.4)/19.9 (14.7–23.3)	379.6 (314.3–475.2)/N/A
Kotwal et al. (2015) [18]	India	No	Not Available	No	No	8.19 ± 2.6/10.99 ± 2.15	10.32 ± 2.43/32 ± 2.8	279.6 ± 20.72/520 ± 38.8
van Dijk et al., B-PROOF (2015) [41]	The Netherlands	No	Placebo 74.2 (6.4) Treatment 74.0 (6.6)	No	No	Placebo: 14.5 (13.0–16.7)/14.3 (12.4–17.0) Treatment: 14.3 (13.0–16.5)/10.3 (8.9–12.0)	Placebo: 18.8 (14.7–21.2) Treatment: 18.7 (14.7–24.4)	Placebo: 265.9 (203.9–343.4)/NA Treatment: 267.3 (212.9–341.2)/NA
Bostom et al. (2011) [21]	US, Canada, Brazil	Yes	52 ± 9.4	No	Stable kidney transplant recipient ***	16.4 ± 1.3 (overall baseline)/11.8 ± 3.8 (HD post-treatment); 15.9 ± 5.5 (LD post-treatment)	N/A	N/A
SEARCH Trial (2010) [25]	UK	No	64.2 ± 8.9	Yes, history of MI	14% with GFR < 60	13.5 ± 4.8/Reduced by 3.8 ± 0.1	7.4 ± 4.6/Increased by 16.2 ± 0.5	388 ± 240/Increased by 625 ± 19
Heinz et al. (2010) [26]	Germany	No	61 ± 13	No	ESRD on dialysis	Placebo: 28.2 (13.0–62.0)/22.3 (9.8–54.1) Treatment arm: 28.7 (16.5–69.4)/18.8 (7.2–33.6)	Placebo 11.8 (5.7–61.4)/ 15.0 (8.2–83.6) Treatment arm: 12.7 (5.7–118.5)/81.8 (34.0–117.4)	Placebo: 288 (140–690)/399 (227–731) Treatment arm: 279 (72–999)/407 (163–1058)
VITATOPS (2010) [24]	20 countries	Variable	62.6 ± 12.5	Yes, recent stroke or TIA	No	14.3 ± 8.5/10.5 ± 4.9	922 ± 476/NA	322 ± 182/NA
Galan et al. (2010) [22]	France	No	60.4–60.9	Yes, history of MI, unstable angina or stroke	No	Placebo: 12.6 (10.4–15.5)/14.5 (12.4–18.3) B vitamins group: 13.0 (11.2–16.0)/11.4 (9.9–14.4)	Placebo: 7.0 (5.3–9.0)/6.5 (5.2–8.1) B vitamins group: 6.7 (5.2–8.5)/15.4 (11.4–19.4)	Placebo: 376 (306–474)/370 (312–469) B vitamins group: 359 (298–455)/497 (390–615)
House et al. (2010) [23]	Canada	Yes	60	Variable	Variable	Placebo: 16.4 ± 5.4/increased by 2.6 ± 0.4 B vitamin group: 14.7 ± 4.9/decreased by 2.2 ± 0.4	Placebo 15 ± 15/NA B vitamin group: 16 ± 37/NA	Placebo: 474 ± 286/NA B vitamin group: 412 ± 193/NA
Ebbing et al., (WENBIT 2008) [28]	Norway	No	61.7	Yes, stable angina/double- or triple-vessel disease/ACS	No	10.8 ± 4.5/7.6 ± 2.2	N/A	N/A
Albert et al. (2008) [27]	US	Yes	Placebo: 62.8 ± 8.8 Treatment: 62.8 ± 8.8	No (either a history of CVD or three or more coronary risk factors)	No	18.5% less than after treatment/9.8	0 subjects > 40 ng/mL/49.3% subjects > 40 ng/mL	N/A
Jamison et al. (2007) [29]	US	Yes	Placebo: 66.2 ± 11.5 Treatment: 65.4 ± 12.0	No	CKD (eGFR ≤ 30 mL/min) or ESRD	Placebo 22.3 (18.7–26.9)/21.6 (18.1–26.9) Treatment: 22.5 (18.9–27.3)/16.5 (13.8–20.1)	Placebo 15.5/16.5 Treatment: 15.7 (9.6–25.0)/ 2019 (501–4067)	N/A
Bønaa et al. (NORVIT, 2006) [31]	Norway	No	FA/B12/B6: 63.6 ± 11.9 FA/B12: 63.2 ± 11.6 B6: 62.5 ± 11.7 Placebo: 62.6 ± 11.4	Yes, acute MI within 7 days	No	FA/B12/B6: 13.1 ± 5.0/9.5 ± 3.6 FA/B12: 12.9 ± 4.3/9.8 ± 4.0 B6: 13.3 ± 6.1/13.3 ± 5.4 Placebo: 13.2 ± 5.2/13.6 ± 6.2	FA/B12/B6: 13.1 ± 27.5/61.8 ± 31.7 FA/B12: 11.7 ± 28.4/70.4 ± 36.4 B6: 9.4 ± 6.6/10.4 ± 9.6 Placebo: 9.6 ± 6.0/13.1 ± 14.5	FA/B12/B6: 388 ± 161/638 ± 370 FA/B12: 400 ± 311/ 648 ± 414 B6: 388 ± 167/398 ± 320 Placebo: 383 ± 182/390 ± 171
Zoungas et al. (ASFAST, 2006) [32]	Australia, New Zealand	No	56	Yes, CVD history	All patients had CKD	Estimated difference in mean tHcy between treatment groups at 48 months 4.7 (95% CI: 9.4 to 0.1; *p* 0.05)	Increased 3-fold in FA group	NA
HOPE 2 Trial (2006) [30]	Canada, US, Brazil, Western Europe, and Slovakia	Yes for US and Canada only	Placebo 68.9 ± 6.8 Treatment 68.8 ± 7.1	Yes, history of vascular disease	No	Placebo 12.0 ^/12.9 Treatment 12.1 ^/9.7	Placebo: 28 ^/23 ^ (at 2 years) Treatment: 28 ^/43 ^ (at 2 years)	Placebo 300 ^/300 ^ Treatment 300 ^/780 ^
Wrone et al. (2004) [35]	US	Yes	59.51–61.30	Variable	Yes, on dialysis	1 mg group: 34.71 ± 20.22/decreased by 3.7 5 mg group 30.62 ± 14.36/decreased by 4.3 15 mg group: 33.52 ± 26.61/decreased by 10.2	1 mg group: 45.91 ± 29.87/NA 5 mg group: 47.16 ± 34.26/NA 15 mg group 49.04 ± 34.85/NA	1 mg group: 503.21 ± 314.47/NA 5 mg group: 514.51 ± 322.93/NA 15 mg group: 518.16 ± 548.32/NA
Toole et al. (VISP, 2004) [36]	US, Canada, Scotland	Yes for US and Canada only	LD: 66.2 ± 10.8 HD: 66.4 ± 10.8	Yes, nondisabling cerebral infarction	No	13.4 at each group/11 (HD); 13.4 (LD)	26 ^/80 ^ (HD); 26 ^ (LD)	370 ^/700 ^ (HD); 400 ^ (LD)
Liem et al. (2004) [37]	The Netherlands	No	59	Yes, history of acute MI	No	N/A	N/A	N/A
Liem et al. (2003,2005) [33,41]	The Netherlands	No	FA 64.9 ± 9.9 Control 65.5 ± 9.7	Unspecified, stable CAD	No	FA12.0 ± 4.8/9.4 ± 3.5 Control 12.2 ± 3.8/NA	FA 17 ± 7/33 ± 6 Control 15 ± 5/NA	FA 286 ± 129/NA Control 294 ± 162/NA
** Trials on Venous Thrombotic Events **								
Shu et al (2017) [38]	China	No	64.7±2.5	Yes, homocysteine cerebral infarction with DVT	No	30.13 ± 1.84/ 10.45 ± 2.62	7.25 ± 2.35/ 15.13 ± 5.23	323.52 ± 93.76/ 645.92 ± 102.48
den Heijer, et al VITRO (2007) [39]	The Netherlands, Italy, Austria	No	Hyperhomocystenie mia group: 56.8 * Normohomocysteeinemia group: 47.3 **	Yes, history of DVT and PE	No	Hyperhomocysteniemia group: 15.1/8.1–8.9	NA	NA

Note: This table only includes the primary study and the post hoc or subgroup analysis of the primary studies were not included. FA: folic acid group; B_6_: vitamin B_6_ group; B_12_: vitamin B_12_ group. HD: high-dose group; LD: low-dose group. * Decrease in high-dose group at 2 years, visually estimated from graph. ** Weighted average of the means and a pooled variance for the standard deviation. *** 6 months post kidney transplant. ^ Estimated from the figures in the article. Plus-minus values are means ± SD; other values are median (interquartile range). Notably, Heinz et al.’s data were reported as median [5th to 95th percentile].

## Supplementary Materials

The following supporting information can be downloaded at: https://www.mdpi.com/article/10.3390/nu17071122/s1, Table S1: Summary of RCTs Studying the Effect of Vitamin B Supplements on the Risk of Thrombotic Events (January 1996–February 2025).
