# Supplementary material for: Effects of B Vitamins on Homocysteine Lowering and Thrombotic Risk Reduction—A Review of Randomized Controlled Trials Published Since January 1996
Source: Nutrients. 2025 Mar 24;17(7):1122. doi: 10.3390/nu17071122 (PMC11990291; doi:10.3390/nu17071122)
Supplement: Supplementary file 1 [file nutrients-17-01122-s001.zip › nutrients-3485668-supplementary.pdf]

Table S1: Summary of RCTs Studying the Effect of Vitamin B Supplements on the Risk of Thrombotic Events (January 1996 – February 2025).

Study	Sample Size and Population	Intervention and Comparison	Duration	Relevant Outcomes
**Trials on Arterial Thrombotic Events**				
Wu et al CSPPT trial 2021[13]	20424 hypertensive adults without a history of stroke or MI	Enalapril 10mg and folic acid 0.8mg (single pill) daily vs Enalapril 10mg daily alone	4.5 years	Risk of first stroke:<br>Males with baseline albumin-corrected serum calcium ≥2.43 mmol/L: 3.0% in the enalapril-folic acid group vs 6.5% in enalapril group (adjusted HR, 0.49; 95% CI: 0.35, 0.68). Risk reduction is 51%.<br><br>Males with baseline albumin-corrected serum calcium<2.43 mmol/L: 2.6% in the enalapril-folic acid group vs 2.3% in enalapril group (adjusted HR, 1.12; 95% CI: 0.76, 1.63). Folic acid had no significant effects.<br><br>Females with baseline albumin-corrected serum calcium ≥2.43 mmol/L: 2.6% in the enalapril-folic acid group vs 2.6% in enalapril group (adjusted HR, 0.99; 95% CI: 0.73,1.34). Folic acid had no significant effects.<br><br>Female with baseline albumin-corrected serum calcium<2.43 mmol/L. 2.5% in the enalapril-folic acid group vs 3.1% in enalapril group (adjusted HR, 0.8; 95% CI: 0.58, 1.11). Folic acid had no significant effects.
Oliari Araghi et a l, B-PROOF trial extended follow-up 2021[14]	1298 patients with aged≥ 65 with an elevated Hcy level (12-50 μmol/l)	Folic acid (400 μg daily) and vitamin B_{12} (500 μg daily) vs placebo	5-7 years	Risk of any type of CVD:<br>130 events in treatment group vs 120 in placebo group; OR 1.05, 95% CI 0.80-1.39, p=0.72;<br>Sex was a significant modifier in the intervention effect on any type of CVD (p-for interaction = 0.03)<br>For women: OR=1.53, 95% CI 0.99-2.35;<br>For men: OR=0.81, 95% CI 0.56-1.17 for any type of CVD<br><br>Risk of MI:<br>20 events in the treatment group vs 18 in the placebo group; OR 1.08, 95% CI 0.56-2.05, p=0.84;<br><br>Risk of cerebrovascular disease:<br>27 events in the treatment group vs 30 in the placebo group; OR 0.85; 95% CI 0.50-1.45.
Kong at al	10789 hypertensive adults without a	Enalapril 10 mg-Folic acid 0.8mg (single	4.5 years (median)	Risk of first stroke:

Post-hoc analysis of CSPPT 2018[15]	history of stroke or MI	pill) daily vs Enalapril 10mg daily alone		Low platelet count group (<210 × 10^9/l): 1.9% in enalapril-folic acid group vs 4.6% in enalapril group (P <0.001)<br>Medium to high platelet count group (≥210 × 10^9/l): 3.3% in enalapril-folic acid group vs 3.7% in enalapril group (P= 0.410)<br><br>Risk of ischemic stroke risk:<br>Low platelet count group (<210 × 10^9/l): 1.6% in enalapril-folic acid group vs 4.1% in enalapril group (P<0.001);<br>Medium to high platelet count group (≥210 × 10^9/l): 2.9% in enalapril-folic acid group vs 3.1% in enalapril group (P= 0.591)<br><br>Risk of hemorrhagic stroke:<br>Low platelet count group (<210 × 10^9/l): 0.2% in enalapril-folic acid group vs 0.5% in enalapril group (P= 0.243);<br>Medium to high platelet count group (≥210 × 10^9/l): 0.49% in enalapril-folic acid group vs 0.5% in enalapril group (P= 0.302)<br><br>Subgroup analysis of joint effect of platelet and homocysteine level:<br>Risk of first stroke risk:<br>Low platelet count group (<210 × 10^9/l) and low tHcy (<15 umol/L):<br>1.9% in enalapril-folic acid group vs 4.2% in enalapril group<br><br>Low platelet count group (<210 × 10^9/l) and high tHcy (<15 umol/L):<br>1.8% in enalapril-folic acid group vs 5.6% in enalapril group<br><br>Medium to high platelet count group (<210 × 10^9/l) and low tHcy (>15µmol/L):<br>3.0% in enalapril-folic acid group vs 3.3% in enalapril group<br><br>Medium to high platelet count group (<210 × 10^9/l) and high tHcy (>15µmol/L):<br>4.1% in enalapril-folic acid group vs 4.7% in enalapril group
Zhao at al<br><br>Post-hoc analysis of CSPPT 2017[16]	20424 hypertensive adults without a history of stroke or MI	Enalapril 10 mg-folic acid 0.8 mg (single pill) daily vs Enalapril 10 mg daily alone	4.5 years (median)	There was a s significant MTHFR gene–homocysteine interaction on first stroke<br><br>Risk of first stroke:<br>CC/CT MTHFR genotype: folic acid supplementation reduced stroke risk by 15% (HR, 0.85; 0.70–1.02);

				TT MTHFR genotype: folic acid supplementation reduced stroke risk by 30% (HR, 0.70; 0.51–0.95)<br><br>Among patients with CC/CT MTHFR genotype, folic acid supplementation significantly reduced stroke risk in the patients high tHcy level (tHcy>13.5μmol/L) (HR, 0.73; 95% CI, 0.55–0.97)<br><br>Among patients with TT MTHFR genotype, folic acid supplementation significantly reduced stroke risk in the patients with lowest tHcy level (tHcy<12.8μmol/L) (HR, 0.44; 95% CI, 0.24–0.79)
Huo et al<br><br>The CSPPT trial 2015[17]	20424 hypertensive adults without a history of stroke or MI	Enalapril 10mg - folic acid 0.8mg (single pill) daily vs Enalapril 10mg daily alone	4.5 years (median)	Risk of first stroke: 2.7% in enalapril-folic acid group vs 3.4% in enalapril group (HR, 0.79 [95% CI, 0.68-0.93]; *P* =0 .003)<br><br>Risk of first ischemic stroke: 2.2% in the enalapril–folic acid group vs 2.8% in the enalapril group; HR, 0.76; 95% CI, 0.64-0.91; *P* = 0.002)<br><br>Risk of composite of first stroke and all-cause mortality: 5.4% in the enalapril–folic acid group vs 6.2% in the enalapril group (HR, 0.86; 95% CI, 0.77-0.97; *P* = 0.01))<br><br>Risk of composite cardiovascular events: 3.1% in the enalapril–folic acid group vs 3.9% in the enalapril group; HR, 0.80; 95% CI, 0.69-0.92; *P* = 0.002)<br><br>Risk of hemorrhagic stroke: 0.56% in the enalapril–folic acid group vs 0.60% in the enalapril group; HR, 0.93; 95% CI, 0.65-1.34; *P* = 0.71)<br><br>Risk of MI: 0.24% in the enalapril–folic acid group vs 0.23% in the enalapril group; HR, 1.04; 95% CI, 0.60-1.82; *P* =0 .89)<br><br>Risk of all-cause deaths (2.9% in the enalapril–folic acid group vs 3.1% in the enalapril group; HR, 0.94; 95% CI, 0.81-1.10; *P* = 0.47)
Kotwal et al, 2015[18]	6000 Armed Forces personnel in the high altitude area	Vitamin B_{12} 1000μg, B_6 3 mg and folic 5mg daily vs no treatment	2 years	B vitamins were effective in reducing Hcy, PAI 1, fibrinogen levels and increasing NO levels at 1 year and reducing the incidence of thrombosis at 2 years<br><br>At 2 years,<br>Incidence of total thrombotic events: 8.33% (5 events) in the treatment group vs 28.33% (17 events) events in the non-treatment group (relative risk = 0.29 (95% CI , 0.11-0.80)

				Incidence of DVT/abdominal vein thrombosis: 2 events in the treatment group vs 6 events in the non-treatment group<br><br>Incidence of PE with or without DVT: 0 events in the treatment group vs 3 events in the non-treatment group<br><br>Incidence of stroke /cortico venous thrombosis: 0 events in the treatment group vs 3 events in the non-treatment group<br><br>Incidence of CAD/MI in age <45 years:1 events in the treatment group vs 5 events in the non-treatment group<br><br>Incidence of pulmonary arterial hypertension: 2 events in the treatment group vs 0 events in the non-treatment group
Arshi et al Post hoc analysis of VISP 2015[19]	3680 patients with non-disabling post-ischemic stroke	High dose group (vitamin B_6 25 mg , vitamin B_{12} 0.4 mg, and folic acid 2.5 mg daily) vs Low dose group (vitamin B_6 200 µg, vitamin B_{12} 6 µg, and folic acid 20 µg daily)	2 years	Risk of stroke:<br>Patients with concurrent antiplatelets therapy: high-dose B vitamins therapy was associated with higher stroke risk (HR, 1.43; 95%CI, 1.02–2.01)<br>Patients without antiplatelet therapy: no significant difference between high-dose and low-dose groups (HR, 0.86; 95%CI, 0.62–1.19).<br><br>Risk of stroke, MI and vascular death:<br>Patients with concurrent antiplatelets therapy: no significant difference between high-dose and low-dose groups (HR 0.18; 95% CI 0.90-1.54)<br>Patients without antiplatelet therapy: no significant difference between high-dose and low-dose groups (HR 0.9; 95% CI 0.7-1.17)
Hankey et al, VITATOPS post-hoc analysis 2012 [20]	8164 patients with recent stroke or TIA(within the past 7 months)	Folic acid 2 mg, vitamin B_6 25 mg, and vitamin B_{12} 0·5 mg daily vs placebo	3.4 years	Risk of primary endpoints of stroke, MI, or death from vascular causes:<br>15% patients from the B vitamins group and 17% patients from the placebo group (risk ratio 0·91, 95% CI 0·82 to 1·00, p=0·05; absolute risk reduction 1·56%, 95% CI −0·01 to 3·16);<br><br>Risk of MI and stroke based on baseline antiplatelet treatment:<br>Risk of stroke:<br>With any antiplatelet therapy: 8.9% vs 9.0%, HR 1.03, 95% CI 0.87-1.12;<br>Without any antiplatelet therapy: 8.9% vs 12.2%, HR 0.65, 95% CI 0.46-0.91; p for interaction=0.01;<br><br>Risk of MI:

				With any antiplatelet therapy: 3% vs 2.9%, HR 0.97, 95% CI 0.72-1.31;<br>Without any antiplatelet therapy: 2.5% vs 2.6%, HR 0.85, 95% CI 0.45-1.79; p for interaction=0.96
Bostom et al. 2011[21]	4110 patients with stable kidney transplant recipients	High-dose: folic acid 5.0 mg, vitamin B_6 50 mg , and vitamin B_{12} 1.0 mg daily;<br>Low-dose: vitamin B_6 1.4 mg and vitamin B_{12} 2.0 μg daily	4 years	Risk of primary outcomes of CVD events:<br>Primary outcome: HR 0.99; 95% CI, 0.84–1.17, p=0.93.<br><br>Risk of individual endpoint:<br>CVD death: 75 events(3.6%) in high-dose group vs 91(4.4%) in low-dose group; HR 0.84; 95% CI, 0.62–1.15, p=0.28;<br>Fatal/Nonfatal stroke: 35 events(1.7%) in high-dose group vs 32(1.6%) in low-dose group; HR 1.12; 95% CI, 0.69, 1.81, p= 0.64;<br>Fatal/Nonfatal MI: 90 events(4.4%) in high-dose group vs 86(4.2%) in low-dose group; HR 1.08; 95% CI, 0.80, 1.45, p=0.61
Galan et al. 2010[22]	2501 patients with history of MI, UA or ischemic stroke	5-methyltetrahydrofolate (5-methyl-THF) 560 μg, vitamin B_6 3 mg, and vitamin B_{12} 20 μg daily vs placebo	4.7 years	Risk of non-fatal MI, stroke, or death from CV causes:<br>75 patients in the treatment group (6.0%) vs 82 in the placebo group (6.5%), HR, 0.90, 95%CI 0.66-1.23, P=0.50.<br><br>Risk of individual endpoint:<br>Compared to placebo group, the patients from treatment group had fewer stroke events (1.7% vs 2.9%; HR 0.57; 95% CI 0.33 -0.97; P=0.04);<br><br>The treatment of B vitamins did not decrease the risk of nonfatal MI (2.3% vs 2.5%; HR 0.88; 95% CI 0.53-1.46; P=0.61)<br><br>And a significantly higher risk of death from any cause (5.8% vs 3.6%; HR 1.55; 95% CI 1.07-2.25); P=0.02).
House et al. 2010[23]	238 patients with diabetes and diagnosed diabetic nephropathy	Folic acid 2.5 mg , vitamin B_6 25 mg, and vitamin B_{12} 1 mg daily vs placebo	2.7 years	Risk of primary endpoint of change in radionuclide GFR:<br>The mean GFR decreased significantly in patients from the treatment group (mean decrease of 16.5 mL/min/1.73 m^2) than those from placebo group (10.7 mL/min/1.73 m^2) (mean difference of −5.8; 95% CI, −10.6 to −1.1; P=0.02).<br><br>Risk of MI, stroke, revascularization, and all-cause mortality:<br>There were more events in B vitamins group (23.5% vs 14.4%; HR 2.0; 95%CI,1.0-4.0; P=0.04)<br><br>Risk of stroke:

				7.2% in B vitamins group vs 1.3 % in placebo group, HR 6.6 (0.8-54.4); P=0.08<br><br>Risk of MI:<br>7.8% in the vitamin B group vs 4.6% in the placebo group. HR2.1 (0.6-6.9); P =0.23
VITATOPS trial 2010 [24]	8164 patients with recent stroke or TIA(within the past 7 months)	Folic acid 2 mg, vitamin B_6 25 mg , and vitamin B_{12} 0.5 mg daily vs placebo	3.4 years	Risk of composite primary endpoint of non-fatal stroke, non-fatal MI, or vascular death:<br>Occurred in 616 (15%) in the B vitamins group (4·3% per year) and in 678 (17%) in the placebo group (4·8% per year; RR 0·91, 95% CI 0·82-1·00; p=0·05; absolute risk reduction 1·56%, 95% CI −0·01 to 3·16).<br><br>Risk of individual endpoint:<br>Compared with placebo, treatment with B vitamins was not associated with a significant reduction in the RR for non-fatal or fatal stroke (9% vs. 10%, p=0·25), non-fatal or fatal MI (3% vs 3%, p=0·86), or death from any cause (p=0·49) but was associated with a significant reduction in death from vascular causes 8% vs 9% (p=0·04)
SEARCH trial 2010[25]	12064 survivors of MI	Folic acid 2 mg and vitamin B_{12} 1 mg daily vs placebo	6.7 years	Risk of first major vascular event (including coronary death, MI, or coronary revascularization), fatal or nonfatal stroke, or non coronary revascularization:<br>1537 of 6033 participants (25.5%) allocated folic acid and vitamin B_{12} vs 1493 of 6031 participants (24.8%) allocated placebo (RR 1.04; 95%CI, 0.97-1.12; P=0.28).<br><br>There were no differences on major coronary events (20.4% vs 19.6%; RR, 1.05; 95% CI, 0.97-1.13), nonfatal MI (7.1% vs 7.1%), stroke (RR, 1.02; 95%CI, 0.86-1.21), or non coronary revascularizations RR, 1.18; 95% CI, 0.95-1.46), or any nonfatal or fatal pulmonary emboli (vitamins, 52 [0.9%], vs placebo, 60 [1.0%]).
Heinz et al 2010[26]	650 patients with ESRD	Active treatment: folic acid 5 mg, vitamin B_{12} 50 μg, and vitamin B_6 20 mg given 3 times a week; Placebo: folic acid 0.2 mg , vitamin B_{12} 4 μg, and vitamin B_6 1.0	2.1 years [median]	Risk of total mortality:<br>Experienced in 31% of patients receiving the active treatment and in 28% for placebo group (HR 1.13; 95% CI, 0.85 to 1.50; P=0.51).<br><br>Risk of first CV events: 25% vs 30% of patients experienced any of the fatal or non-fatal CV events in the treatment and placebo group, respectively (HR, 0.80; 95% CI, 0.60 to 1.07; P=0.13).<br><br>Compared to the treatment group with placebo, there were no differences on separate endpoints of MI (6% vs 6%; HR 1.00, p=0.99), stroke (3% vs 5%; HR 0.73,

		mg , given 3 times a week.		p=0.43), or deaths of cardiac causes (11% vs 10%; HR 1.26, p=0.36).
Albert et al 2008[27]	5442 female US health professionals with either a history of CVD or ≥three coronary risk factors	Folic acid 2.5 mg , vitamin B_6 50 mg, and vitamin B_{12} 1 mg daily vs placebo	7.3 years	Risk of primary endpoints of MI, stroke, CABG/PCI, CVD death:<br>226.9/10,000py in the vitamin group compared to 219.2/10,000py in the placebo group; RR=1.03; 95% CI 0.90–1.19, P=0.65;<br><br>Risk of MI: RR=0.87; 95% CI 0.63-1.22;<br>Risk of stroke: RR=1.14; 95% CI 0.82-1.57<br>Risk of any death caused by CVD: RR=1.01; 95% CI 0.76-1.35<br><br>Comparison between patients with prior CVD vs without prior CVD:<br>There was no significant difference between the two groups (P for interaction=0.93)
Ebbing et al WENBIT trial 2008[28]	3096 patients undergoing coronary angiography	Four groups: folic acid 0.8 plus vitamin B_{12} 0.4 mg plus vitamin B_6 40 mg daily; folic acid plus vitamin B_{12} daily; vitamin B_6 daily alone; and placebo.	3.2 years [median]	Risk of primary endpoints of all-cause death, MI, acute hospitalization for USA, and ischemic stroke:<br>FA group vs non-FA group: 14.2% vs 13.1% experienced events, HR 1.09, 95% CI, 0.90-1.32, P=0.36<br>Vitamin B_6 group vs non-B_6 group: 13.0% vs 14.3% experienced events, HR 0.90, 95% CI 0.74-1.09, P=0.28.<br><br>There were no significant differences in treatment response for the separate end points of MI (FA vs non-FA: HR 1.21; 95%CI 0.95-1.56; B_6 vs non-B_6: HR 0.85; 95%CI 0.66-1.09) or any type of stroke (FA vs non-FA: HR 0.72; 95%CI 0.44-1.17; B_6 vs non-B_6: HR 0.87; 95%CI 0.54-1.40).<br><br>Kaplan-Meier curves showed an increased risk of 34% of primary outcomes in FA plus B_{12} group compared to placebo group (95% CI, 1.03-1.75; P=0.03). There were no differences between groups regarding survival analysis (p=0.07).<br><br>Increase of baseline Hcy level by 3 μmol/L was associated with increased risk of primary endpoints: HR 1.07, 95% CI 1.02-1.13, p=0.01.
Jamison et al, 2007[29]	2056 patients with advanced CKD (eCrCl 30 mL/min) or ESRD, and high Hcy levels (≥15 μmol/L).	Folic acid 40 mg, vitamin B_6 100 mg , and vitamin B_{12} 2 mg daily vs placebo	3.2 years [median]	Risk of primary endpoint of all-cause mortality:<br>There were no differences between groups (43% vs 43%; hazard ratio, 1.04; 95%CI, 0.91-1.18)<br><br>Risk of MI:<br>There were 129 events (13%) in the vitamin group vs 150 for placebo (15%), HR=0.86, 95% CI 0.67-1.08;<br><br>Risk of stroke:

				There were 37 events in the vitamin group (4%) vs 41 for placebo (4%), HR=0.90, 95%CI 0.58-1.40
HOPE 2 trial 2006[30]	5522 patients with vascular disease or diabetes	Folic acid 2.5 mg , vitamin B_6 50 mg, and vitamin B_{12} 1 mg daily vs placebo	5 years	Risk of primary endpoints of MI, stroke or death from CV cause:<br>18.8% in the vitamins group and 19.8% for placebo, RR 0.96, 95% CI 0.84-1.07, p=0.41<br><br>Risk of non-fatal stroke:<br>Fewer patients from the treatment group had a stroke (RR=0.75; 95% CI, 0.59-0.97)<br><br>Risk of other endpoints:<br>There were no significant differences on death from CV causes (RR=0.96; 95% CI, 0.81 to 1.13) and MI (RR=0.98; 95% CI, 0.985-1.14)
Bønaa et al NORVIT trial 2006[31]	3749 patients who had had an acute MI within 7 days before randomization	Four groups:<br>G1: folic acid 0.8 mg , vitamin B_{12} 0.4 mg , and vitamin B_6 40 mg daily ;<br>G2: folic acid 0.8 mg and vitamin B_{12} 0.4 mg daily;<br>G3: vitamin B_6 40 mg daily;<br>Vs G4: placebo	3.3 years [median]	Risk of recurrent MI, stroke, and death of CAD causes:<br>Treatment with folic acid plus B_{12} vs no folic acid/B_{12}: no difference between the groups (RR,1.08; 95% CI, 0.93-1.25; P=0.31);<br>Treatment with B_6 vs non-B_6: there was a trend of increase in risk of primary endpoints (RR,1.14; 95% CI, 0.98-1.32; P=0.09);<br>Treatment with folic acid, B_{12} and B_6 vs placebo: there was an increase of risk associated with the treatment (RR,1.22; 95% CI, 1.00-1.50; P=0.05)<br><br>Risk of MI: no difference between the treatment group with FA, B_{12} and B_6 and the placebo group (RR 1.23; 95% CI 0.99-1.52), but there was an increased risk of non-fatal MI in the treatment group (RR1.3; 95% CI, 1.00-1.68, p=0.05);<br><br>Risk of stroke: no difference between the treatment group with FA, B_{12} and B_6 and the placebo group (RR 0.83; 95% CI 0.83-1.83)
Zoungas et al, ASFAST trial 2006[32]	315 patients with ESRD	folic acid 15 mg daily vs placebo	3.6 years [median]	Risk of all MI, stroke or death from cardiovascular cause:<br>8.9 per 100 py in the vitamin group as compared with 10.4 per 100 py in the placebo group, adjusted HR 0.98, 95% CI 0.66-1.47, p=0.94<br><br>Risk of all cardiovascular events: HR 0.95, 95% CI 0.69-1.30, p=0.75<br><br>Risk of coronary event: 23 events in FA group vs 19 events in placebo group;<br>Risk of cerebrovascular event: 8 events in FA group vs 18 events in placebo group;

				Risk of death from any CV causes: 21 events in FA group vs 24 events in placebo group.
Liem et al, 2005 [33]	593 patients with stable CAD	folic acid 0.5 mg vs standard care	3.5 years	Risk of primary endpoints of death and/or any CV event:<br>There was no difference between the two arms for the risk of death and/or CV events (16.3% in the FA group and 19.1% in the control group, RR 0.85; 95% CI 0.56-1.31).<br><br>Risk of primary endpoints for patients with plasma Hcy > 13.7 μmol/L:<br>There was no difference between the two arms: RR 1.37, 95% CI 0.65-2.87.<br><br>Risk of individual endpoints:<br>All-cause mortality: 18 patients (6%) in the FA group vs 26 patients (8.9%) in the control group;<br>ACS: 23(7.7%) patients in the FA group vs 18(6.1%) patients in the control group;<br>Cerebrovascular accident/TIA: 8(2.7%) events in the FA group vs 12(4.1%) events in the control group.
Spence et al,<br>VISP trial-subgroup analysis 2005[34]	2155 patients with non disabling post-ischemic stroke, baseline vitamin B_{12} in between the 25th percentile and the 95th percentile, GFR≥ the 10th percentile	High dose group (vitamin B_6 25 mg , vitamin B_{12} 0.4 mg, and folic acid 2.5 mg daily) vs low dose group (vitamin B_6 200 μg, vitamin B_{12} 6 μg, and folic acid 20 μg daily)	2 years	Risk of the combined endpoint of ischemic stroke, coronary disease, or death: 20% reduction of risk in the high-dose group compared with the low-dose group (HR 0.80; 95% CI, 0.63-1.01; P=0.056);<br><br>Risk of ischemic stroke: no significant difference between the two groups (HR 0.91; 95%CI, 0.67-1.24; P=0.56);<br>Coronary event: no significant difference between the two groups (HR 0.88; 95%CI, 0.61-1.27; P=0.49);
Wrone 2004[35]	510 patients with ESRD on dialysis	1, 5, or 15 mg of folic acid contained in a renal multivitamin	2 years [median]	Risk of primary endpoints of mortality and CV events:<br>There were no differences between groups: 43.7% in 1 mg group, 38.6% in 5 mg group, 47.1% in 15 mg group (log-rank P=0.47);<br><br>Risk of individual endpoints:<br>MI: 4(2.4%), 5(2.8%), 4(2.4%) patients had the outcome in 1, 5, 15 mg arm, respectively;<br>Cerebrovascular accident: 8(4.8%), 10(5.7%), 9(5.4%) patients had the outcome in 1, 5, 15 mg arm, respectively;<br>Death: 56(33.3%), 44(25%), 61(36.7%) patients had the outcome in 1, 5, 15 mg arm, respectively;

				The reverse association between the baseline Hcy levels and risk of primary endpoints:<br>Unlike other studies, this clinical trial found higher concentration of baseline Hcy was associated with lower risk of primary events (Q: quartiles of baseline tHcy; from lowest quartile to highest quartile: 54.5% of Q1 experienced primary endpoints, 41.8% for Q2, 41.2% for Q3, and 34.7% for Q4; log-rank P=0.033).<br>For every 1 mol/L of increase in the tHcy level, the RR for a primary outcome decreased by 1.4% (P=0.0015).
Toole et al, VISP trial 2004 [36]	3680 non-disabling post-ischemic stroke	High dose group (vitamin B_6 25 mg, vitamin B_{12} 0.4 mg, and folic acid 2.5 mg daily) vs low dose group (vitamin B_6 200 µg, vitamin B_{12} 6 µg, and folic acid 20 µg daily)	2 years	Risk of any stroke, CHD event, or death: 18.0% in the high-dose group and 18.6% in the low-dose group, risk ratio of 1.0 (95% CI, 0.8-1.1).<br><br>Risk of ischemic stroke: 9.2% in the high-dose and 8.8% in the low-dose groups (RR=1.0; 95% CI, 0.8-1.3) (P=0.8);<br>Risk of MI/fatal CHD: RR=0.9; 95% CI 0.7-1.2;<br><br>Association between baseline tHcy level and risk of thrombotic events: for low-dose group: a 3 µmol/L lower tHcy level was associated with a 10% lower risk of stroke (P=0.05), a 26% lower risk of CHD events (P=0.001), and a 16% lower risk of death (P=0.001); for high dose group: 2% for stroke (p=0.24), 7% for CHD events (p=0.002), and 7% for death (p=0.001), respectively.
Liem,et al 2004 [37]	283 patients with a total cholesterol >251 mg/dl	Folic acid 5 mg plus fluvastatin 40 mg daily vs fluvastatin 40 mg daily	1 year	Risk of primary endpoints of death, recurrent MI, strokes, and unplanned PCI:<br>These endpoints occurred in 43 patients (30.7%) in the folic acid group vs. 45 patients (31.5%) in the control group (RR, 0.97, p value not reported);<br><br>Risk of individual endpoints:<br>Fatal and non-fatal stroke: 1 patients in the treatment group vs no patient in the control group;<br>Recurrent MI: 6 patients in the treatment group vs 8 patients in the control group;<br>Fatal MI: 2 patients in the treatment group vs 2 patients in the control group.
**Trials on Venous Thrombotic Events**				
Shu et al 2017 [38]	90 patients with homocysteine cerebral infarction	Folic acid 5 mg and vitamin B_{12} 0.25mg daily vs no treatment	3 months	Vitamin B supplements significantly reduced lower limb DVT recurrence rate.<br><br>Rate of recurrent DVT:<br>4.4% in treatment group vs 28.9% in non-treatment group (p<0.05)

				More stable INR, but lower PT and APTT in treatment group
den Heijer, et al The VITRO trial<br><br>2007 [39]	701 patients between 20 to 80 years with a first objectively confirmed DVT/PE	Folic acid 5 mg , cyanocobalamin 0.4 mg, and pyridoxine 50 mg daily vs placebo	2.5 years	Risk of recurrent VTE: the number of recurrent events of VTE was 43 of 353 in the vitamin group (54/1000 py) and 50 of 348 in the placebo group (64/1000 py), HR=0.84 (95% CI, 0.56-1.26)<br><br>For hyperhomocysteinemia group: HR=1.14 (95% CI, 0.65-1.98)<br>For normohomocysteinemia group: HR=0.58 (95% CI, 0.31-1.07).

Hcy, tHcy: homocysteine, total homocysteine; CVD: cardiovascular disease; CV: cardiovascular; MI: myocardial infarction; eGFR: estimated glomerular filtration rate; ESRD: end-stage renal disease; CKD: chronic kidney disease; eCrCl: estimated creatinine clearance; PAI 1: plasminogen activator inhibitor-1; NO: nitric oxide; CAD: coronary artery disease; CABG: coronary artery bypass graft; ACS: acute coronary syndrome; SVT: supraventricular tachycardia; HF: heart failure; DVT: deep vein thrombosis; PE: pulmonary embolism; VTE: venous thrombotic events; FA: Folic acid; MTHFR: methylenetetrahydrofolate reductase; CC/CT genotype: wild type (C), mutated type (T); TT-homozygous genotype, CT-heterozygous genotype, CC-noncarrier; INR, PT, APTT: international normalized ratio, prothrombin time, activated partial thromboplastin time; HR: hazard ratio; CI: confidence interval; RR: risk ratio; py: patient-years.
